# Comment on Abdul Jabbar et al. Air Quality, Pollution and Sustainability Trends in South Asia: A Population-Based Study. *Int. J. Environ. Res. Public Health* 2022, *19*, 7534

**DOI:** 10.3390/ijerph20065075

**Published:** 2023-03-14

**Authors:** Muhammad Bilal, Gerrit de Leeuw, Janet E. Nichol, Max P. Bleiweiss, Leiku Yang, Huabin Chai, Alaa Mhawish, Md. Arfan Ali

**Affiliations:** 1 School of Surveying and Land Information Engineering, Henan Polytechnic University, Jiaozuo 454003, China; muhammad.bilal@connect.polyu.hk (M.B.);; 2 Royal Netherlands Meteorological Institute (KNMI), R & D Satellite Observations, 3730 AE De Bilt, The Netherlands; 3 Aerospace Information Research Institute, Chinese Academy of Sciences (AirCAS), Beijing 100045, China; 4 Department of Geography, School of Global Studies, University of Sussex, Brighton BN1 9RH, UK; 5 Department of Entomology, Plant Pathology, and Weed Science, New Mexico State University, Las Cruces, NM 88003, USA; 6 School of Marine Sciences, Nanjing University of Information Science and Technology, Nanjing 210044, China; 7 Center of Excellence for Climate Change Research/Department of Meteorology, King Abdulaziz University, Jeddah 21589, Saudi Arabia

This comment discusses the use of PM_2.5_ (mass concentration of fine particulate matter with an aerodynamic diameter less than 2.5 microns) data in the recently published article entitled “Air Quality, Pollution and Sustainability Trends in South Asia: A Population-Based Study” by Abdul Jabbar et al. [1]. The authors have used two types of PM_2.5_ data, i.e., PM_2.5_ concentrations and PM_2.5_ exposure data. The source of PM_2.5_ concentration is not explicitly mentioned; however, the article published by Upadhyay et al. [2] is cited in Figure 4. Upadhyay et al. mentioned that PM_2.5_ concentrations were obtained from the ground-based air quality monitoring stations installed at the US embassies in major cities in South Asian countries. These concentrations are limited to specific cities and do not represent country-level air pollution scenarios. The purpose of this comment is not to discuss the PM_2.5_ concentrations provided by the US embassies, but instead to comment on the use of the PM_2.5_ exposure data, which are used in Abdul Jabbar et al.’s paper. These exposure data are obtained from the World Bank database (https://databank.worldbank.org/source/world-development-indicators (accessed on 17 October 2022)). PM_2.5_ exposure (EXP) is related to PM_2.5_ concentrations through Exp = SUM{(Pi/P) × Ci}, where Ci = annual mean PM_10_ or PM_2.5_ concentration in sub-population Pi, P = SUM (Pi), which is the total population in cities with data [3].

It is well established that both PM_2.5_ concentrations [4] and PM_2.5_ exposure [5] significantly increased in Pakistan during the last few decades. However, the PM_2.5_ exposure data reported by Abdul Jabbar et al. [1] do not show substantial variation between 1990 and 2017, and they state that the mean exposure to PM_2.5_ in Pakistan over the period was “steady”. We illustrate the discrepancy in Figure 1, which plots the time series of PM_2.5_ exposure data (2010–2017) obtained from the World Bank database (which are used by Abdul Jabbar et al. [1]) and PM_2.5_ concentrations obtained from both the Copernicus Atmosphere Monitoring Service (CAMS) reanalysis data (2010–2017) and the World Health Organization (WHO) website (https://www.who.int/data/gho/data/themes/air-pollution/who-air-quality-database, accessed on 17 October 2022) (2010–2016).

Pakistan is the second-most polluted country among South Asian countries, as reported by the authors [1]. Therefore, reliable and accurate information is required for policymakers and research scientists to mitigate air pollution problems in Pakistan. Thus, further investigation is required to resolve discrepancies between PM_2.5_ exposure and concentration data from different sources before they can be used in any scientific research or policy application.

## Figures and Tables

**Figure 1:**
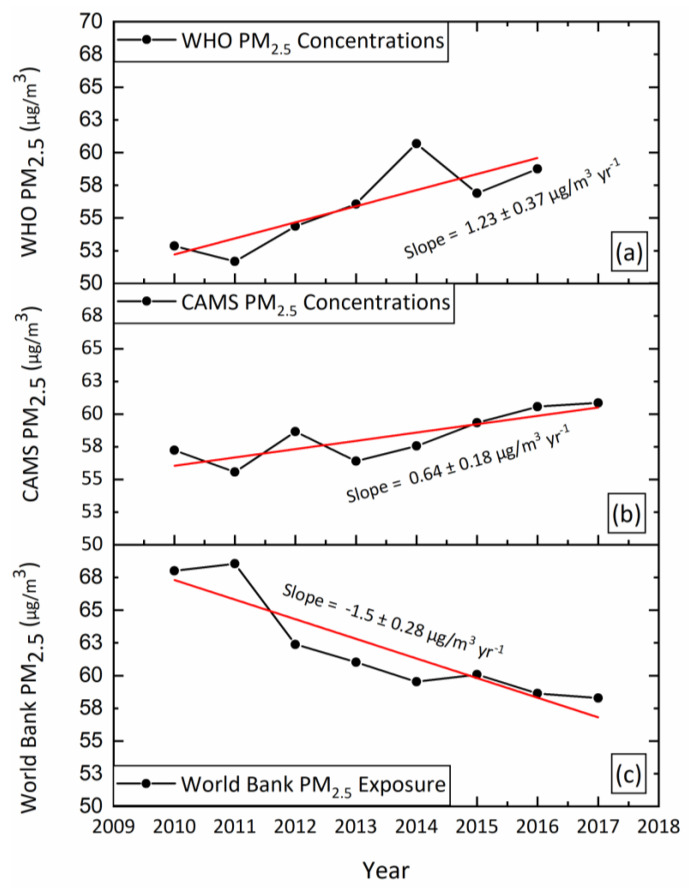
Time series of PM_2.5_ concentrations obtained from (**a**) the WHO database (2010–2016) and (**b**) CAMS reanalysis data (2010–2017). (**c**) PM_2.5_ exposure was obtained from the World Bank database (2010–2017). PM_2.5_ concentrations from the WHO website were not available for 2017.

## Data Availability

The data used in this research are available on the World Bank website (https://databank.worldbank.org/source/world-development-indicators (accessed on 17 October 2022)) and the World Health Organization (WHO: https://www.who.int/data/gho/data/themes/air-pollution/who-air-quality-database (accessed on 17 October 2022)).
